# En Bloc lumpectomy of T12 vertebra for progressive hepatocellular carcinoma metastases following liver transplantation: A case report

**DOI:** 10.1097/MD.0000000000018756

**Published:** 2020-01-10

**Authors:** Jin-Gen Hu, Yang Lu, Xiang-Jin Lin

**Affiliations:** Department of Orthopedics, First Affiliated Hospital, Zhejiang University School of Medicine, Hangzhou 310003, China.

**Keywords:** en bloc, hepatocellular carcinoma, liver transplantation, lumpectomy, spinal metastasis

## Abstract

**Rationale:**

Liver transplantation (LT) is the preferred surgical option for the treatment of early hepatocellular carcinoma (HCC). In contrast, surgical treatment of progressive HCC metastasized to the spine following LT constitutes a considerable challenge. Here, we report the first case of progressive HCC metastasized to the T12 vertebra after local radiotherapy, treated successfully with en bloc lumpectomy following LT for HCC.

**Patient concerns:**

A 40-year-old man who had undergone LT for the treatment of HCC 2 months prior presented to our clinic with symptoms of progressive back pain. Magnetic resonance imagining (MRI) and positron emission tomography (PET) examinations showed a solitary metastasis at T12 without recurrence in the liver or metastasis to other organs.

**Diagnoses:**

The patient was diagnosed with HCC metastasized to the T12 vertebra after liver transplantation.

**Interventions:**

Local radiation therapy of the T12 vertebra was performed; however, the lesion continued to grow one month after irradiation. Accordingly, the patient was treated with en bloc lumpectomy of the T12 vertebra. After surgery, the patient reported significant pain relief. At 11 months post-surgery, a C4 metastasis with spinal cord compression was revealed by MRI. Multiple grafted liver metastases were also detected by ultrasound along with several lung metastases, which were discovered by X-ray. The patient was treated with a pedicle screw system and a mesh cage filled with frozen autografts for C4 metastasis.

**Outcomes:**

The patient died 15 months after liver transplantation due to recurrence in the liver and metastasis to the lung.

**Lessons:**

En bloc lumpectomy may be a viable therapeutic option for patients with progressive solitary spinal metastases after LT refractory to radiotherapy. Use of immunosuppressive therapy after LT may significantly inhibit immune function, making patients more susceptible to HCC recurrence and bone metastasis.

## 1 Introduction

Liver transplantation is an effective treatment for hepatocellular carcinoma (HCC); however, patients remain highly susceptible to metastasis and recurrence after LT, with HCC commonly metastasizing to the grafted liver, lung, and bone, with upwards of 10% of HCC patients exhibiting bone metastases after LT.^[1–3]^ The spine is among the most common sites of bone metastasis, and is associated with a poor prognosis.^[4,5]^ Palliative radiotherapy is a clinically effective option for patients with spinal metastases deemed unsuitable for surgery,^[6]^ though radiotherapy alone is often unable to control tumor progression. While possible, surgical intervention for patients with spinal tumor progression is difficult due to the preceding radiation along with an increased risk of infection. Under these circumstances, patients exhibiting adequate liver function after LT, and considered healthy enough to tolerate surgery are treated by en bloc lumpectomy. Here, we report the clinical outcomes of en bloc lumpectomy for progressive T12 metastasis in a patient previously treated by LT for HCC. This study was approved by the ethics committee of the First Affiliated Hospital of Zhejiang University. The approval number was 2013–212. Informed consent was given.

## 2 Case presentation

**Figure 1:**
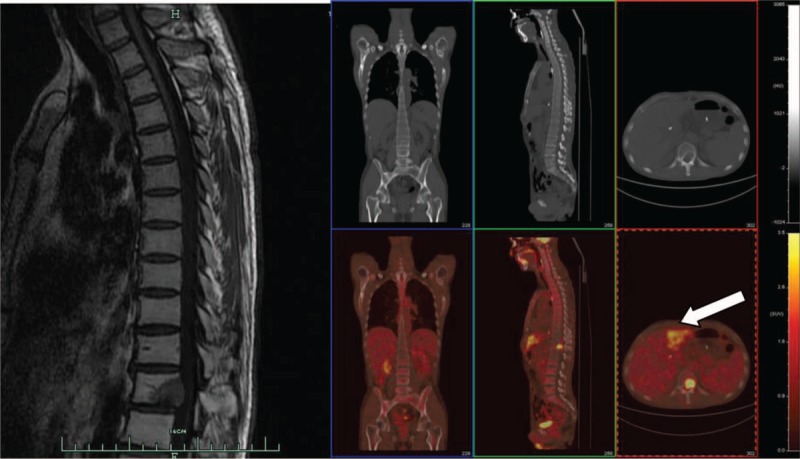
Preoperative radiographic images. Sagittal views of the T1-weighted magnetic resonance images (left) and PET (right) images. Arrows indicate the T12 metastasis and suspected recurrence of hepatocellular carcinoma in the grafted liver.

A 40-year-old man presented to the orthopedics department with complaints of back pain 2 months after undergoing LT for HCC. The patient reported progressive pain without symptoms of spinal cord compression for 1 month prior to admission. Magnetic resonance imaging (MRI) and positron emission tomography (PET) were performed, revealing a solitary metastasis at the T12 vertebra without pathological fractures or spinal cord compression (Fig. 1). PET images revealed highly metabolized areas of the liver, indicating likely recurrence of HCC on the grafted liver.

The patient exhibited poor liver function due to anatomic stenosis after LT, requiring treatment via stent placement surgery. Due to this decrease in liver function, immediate surgical intervention was not chosen as a treatment for metastasis to the T12 vertebra and suspected recurrence of HCC. Instead, radiation therapy of the affected area was performed to alleviate clinical symptoms. After radiation, the patient reported a significant improvement in pain symptoms and alpha-fetoprotein (AFP) levels, a tumor marker for HCC (normal range: < 20 ng/L), quickly returned to normal. However, a month after irradiation, the patient exhibited extremely high serum AFP levels (2526.5 ng/L), indicating a potential recurrence of HCC.

A follow up PET scan showed no metastasis to the grafted liver or other organs, though the T12 lesion was found to have increased in size, indicating progressive disease after radiation therapy. The Tomita and modified Tokuhashi scores for evaluating the prognosis of patients with metastatic spinal tumors were 5 and 11 points, respectively.^[7,8]^

**Figure 2:**
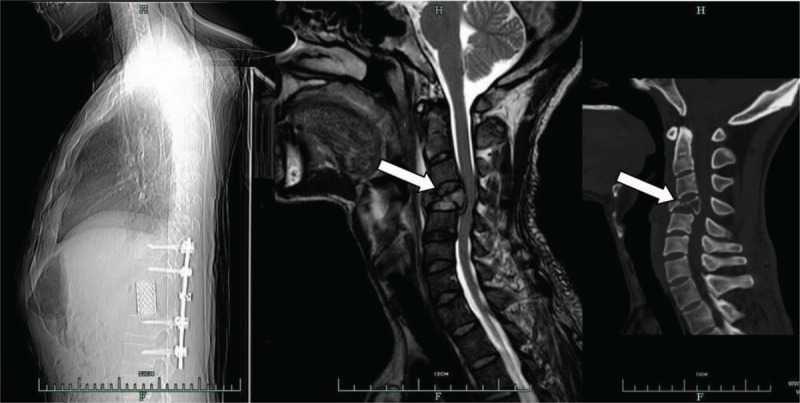
(A) Lateral view of radiographs after total en bloc lumpectomy for T12 metastasis. (B) Sagittal views of the T2-weighted magnetic resonance images and CT images at 3 months post-surgery. Arrows indicate the newly developed metastasis at C4.

The patient was treated via en bloc lumpectomy using the posterior approach. Pedicle screws were placed into the T10, T11, L1, and L2 vertebra bilaterally, and then titanium alloy rods were attached to the screws. After en bloc lumpectomy of the T12 vertebra, anterior reconstruction was achieved through a titanium mesh cage with frozen autografts (Fig. 2A). The operation lasted 5 hours, and intraoperative bleeding was 2000 ml. Pathology after surgery of the T12 vertebra revealed a metastasis of HCC (moderate and low differentiated).

**Figure 3:**
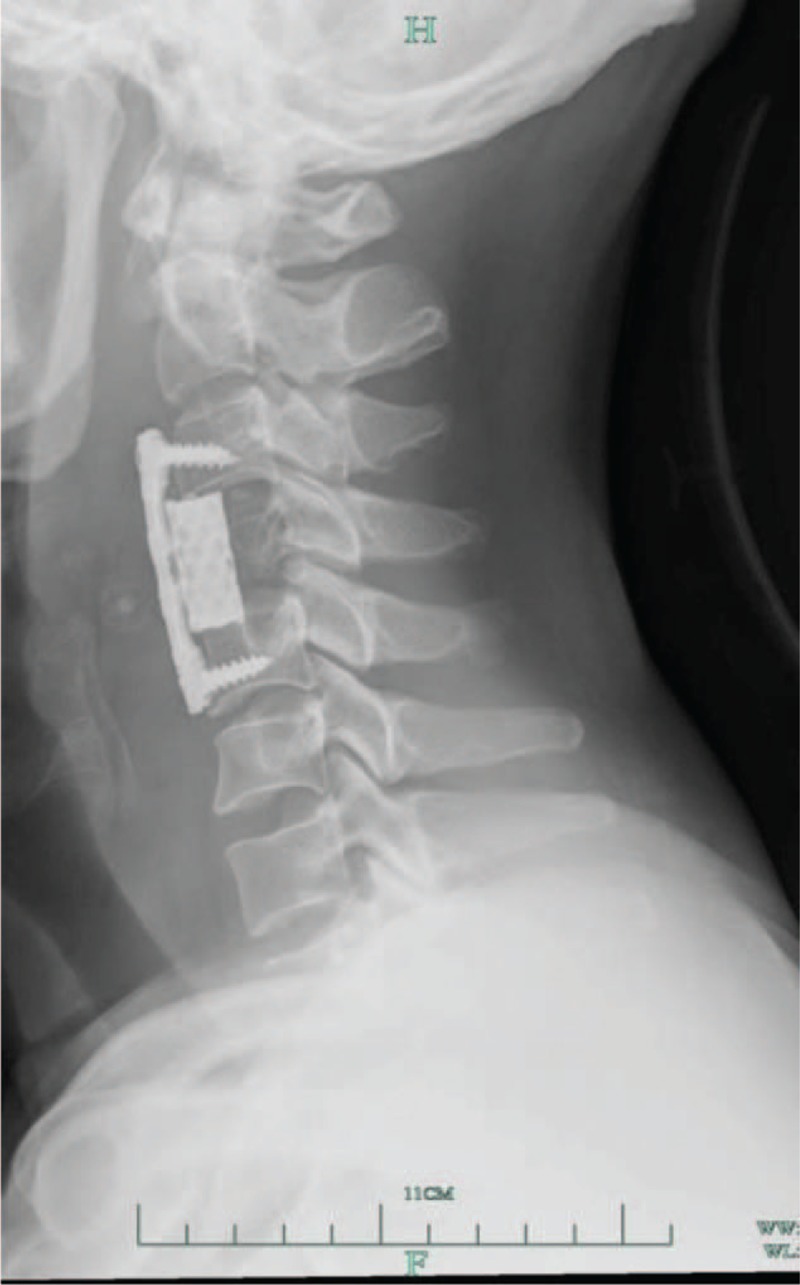
Radiographic images after surgery at C4. Lateral views are shown.

After surgery, the patient developed an infection of the incision, which was treated using anti-inflammatory medications. The infected incision is thought to have been the result of immunosuppressive therapy along with radiotherapy after LT. The patient's AFP levels quickly returned to normal 1 month after surgery; however, this resolution was short lived, with his AFP levels increasing rapidly again 11 months post-surgery, with MRI and CT images indicating metastasis to the C4 vertebra (Fig. 2B). At the same time, multiple grafted liver metastases were detected by B-ultrasound and numerous lung metastases were discovered by X-ray. The patient was treated via resection of the C4 vertebra using an anterior approach. Following resection, anterior reconstruction was achieved using a titanium alloy plate system and a mesh cage filled with frozen autografts (Fig. 3) in accordance with patient preferences. His AFP levels decreased to normal after second surgery, though the patient ultimately died 15 months after liver transplantation due to recurrence in the grafted liver and metastasis in the lung.

## 3 Discussion

The primary therapeutic options for spinal metastatic tumors are typically radiotherapy and surgical intervention. In this study, en bloc lumpectomy technique was performed without fatal complications, and the patient lived longer than initially predicted before en bloc lumpectomy resection. Given the nature of spinal tumors, most treatment plans avoid the use of surgery when predicted survival times are short. To determine the most appropriate treatment course, a variety of scoring systems are used, including the Tomita, Katagiri, and modified Tokuhashi scores, which are used to predict survival and treatment outcomes in patients with spinal metastases.^[7–9]^ However, many of these scoring systems may be inappropriate, as they have yet to be adapted to reflect some of the more recent advances in cancer treatment (i.e., organ transplantation and targeting drugs), which have had a profound effect on the survival of patients with spinal metastases. Moreover, these scoring systems do not include a patient's prognostic score in the case of liver transplantation. Previously, our group showed that the median survival time after bone metastases (BM) in LT (16 cases) and non-LT (27 cases) patients was 20 months and 11 months, respectively. This did not mean that the LT patients have a longer median survival than the non-LT patients.^[10]^ These outcomes are, instead, a reflection of the nature of the patient populations, with LT patients typically diagnosed with earlier stages of HCC, with better intrahepatic tumor control, without extension into soft tissue, compared to non-LT patients.^[10]^

The patient in our study had already undergone radiotherapy for metastasis to the T12 vertebra, which showed an initial improvement in symptoms, followed by a rapid expansion of the metastasis thereafter. Given this initial diagnosis, the patient was predicted to have a very short life expectancy and an overall poor quality of life; however, the patient ultimately lived well beyond the predicted 6- to 12-month survival time.

The origin of metastases in this patient was HCC, which can be managed by multi-modality therapy; for example, LT was used as a potential curative option in this patient. Our current data suggest that patients with metastasis to the T12 vertebra can live longer than currently predicted using available scoring systems when treated using aggressive radiation therapy. Given the advancements in HCC therapy, improved outcomes for procedures such as en bloc lumpectomy may be possible.^[11–13]^ In our hospital, the clinical indications for en bloc lumpectomy surgery for spine metastases require consideration of multiple factors: age, comorbidities, affected spine location, size of HCC, other organ metastases, liver function, and various types of differentiation of HCC.

Despite the potential benefits of en bloc lumpectomy, excision of invasive metastases comes with significant risk, including an increased incidence of complications, spinal instability, and infection. The incidence of postoperative complications in patients with metastatic spinal tumors has been reported at about 20%.^[14]^ Our patient also experienced a significant, though non-life-threatening postoperative infection due to the invasive nature of en bloc lumpectomy and low immune function after LT.

Studies have shown that bone is the third largest extrahepatic metastatic site after grafted liver and lung (18%–33%) in patients undergoing LT for HCC.^[1,15–17]^ A subsequent study identified several clinical characteristics and prognostic factors for bone metastasis after LT for HCC. Among the most commonly affected sites in these patients was that of thoracic vertebrae, affecting 15.1% of all patients.^[18]^ The median survival time in this cohort of patients was 8.6 months, with liver failure or tumor progression as the most common cause of death.

In our case, metastases were found to have spread to the grafted liver, lung, and bone. Among the affected sites, the bone metastasis responded poorly to irradiation, with the bone lesion continuing to grow larger over time. Despite aggressive treatment for the spinal metastasis, additional metastases on the grafted liver and lung persisted, resulting in progressive decompensation of liver function and respiratory failure due to lung metastases, with the patient ultimately succumbing to disease 15 months after LT.

In this patient, bone metastasis appeared after LT, suggesting 2 potential options. The first possibility is that bone metastasis occurred during liver transplantation, with the current diagnosis methods simply unable to detect it. The second option is that immunosuppression after LT can result in insufficient immune function, which can make HCC more prone to recurrence and bone metastasis.

LT patients usually receive immunosuppressive therapy,^[19,20]^ which can accelerate tumor recurrence and metastasis. There is currently no reliable conclusion on the impact of any specific immunosuppressant on the recurrence of HCC; however, the immunosuppressive load seems to play a decisive role.^[21–22]^ The current trend is to minimize the immunosuppression load in consideration of the patient's intrinsic immune status,^[23–25]^ which can increase patient and graft survival. This type of therapy typically needs to be supplemented by adjuvant chemotherapy to enable immunosuppression minimization.^[26]^

Previous reports have examined the use of en bloc lumpectomy for the treatment of spinal metastasis of HCC arising after LT. Based on our experience, we would recommend that only patients with suitable liver function after LT be considered for any surgical intervention, consistent with previous findings.^[27]^ The case presented here suggests that en bloc lumpectomy may be a viable therapeutic option for patients with progression of solitary spinal metastases after LT.

## Acknowledgments

We thank Department of Hepatobiliary Pancreatic Surgery, First Affiliated Hospital, Zhejiang University School of Medicine for providing clinical data of primary tumors.

## Author contributions

**Conceptualization:** Jin-Gen Hu, Yang Lu, Xiang-Jin Lin.

**Data curation:** Jin-Gen Hu, Yang Lu, Xiang-Jin Lin.

**Formal analysis:** Jin-Gen Hu, Yang Lu, Xiang-Jin Lin.

**Funding acquisition:** Jin-Gen Hu, Yang Lu, Xiang-Jin Lin.

**Investigation:** Jin-Gen Hu, Yang Lu, Xiang-Jin Lin.

**Methodology:** Jin-Gen Hu, Yang Lu, Xiang-Jin Lin.

**Project administration:** Yang Lu.

**Software:** Jin-Gen Hu, Yang Lu.

**Supervision:** Yang Lu.

**Validation:** Jin-Gen Hu, Yang Lu.

**Visualization:** Jin-Gen Hu, Yang Lu, Xiang-Jin Lin.

**Writing – original draft:** Jin-Gen Hu, Yang Lu, Xiang-Jin Lin.

**Writing – review & editing:** Jin-Gen Hu, Yang Lu.
